# Active Efflux Leads to Heterogeneous Dissipation of Proton Motive Force by Protonophores in Bacteria

**DOI:** 10.1128/mBio.00676-21

**Published:** 2021-07-13

**Authors:** Dai Le, Ekaterina Krasnopeeva, Faris Sinjab, Teuta Pilizota, Minsu Kim

**Affiliations:** a Department of Physics, Emory University, Atlanta, Georgia, USA; b Graduate Division of Biological and Biomedical Sciences, Emory University, Atlanta, Georgia, USA; c Centre for Synthetic and Systems Biology, School of Biological Sciences, University of Edinburgh, Edinburgh, United Kingdom; University of Pittsburgh

**Keywords:** bacterial physiology, single-cell microscopy, cell-to-cell heterogeneity, efflux pumps, proton motive force, protonophore

## Abstract

Various toxic compounds disrupt bacterial physiology. While bacteria harbor defense mechanisms to mitigate the toxicity, these mechanisms are often coupled to the physiological state of the cells and become ineffective when the physiology is severely disrupted. Here, we characterized such feedback by exposing Escherichia coli to protonophores. Protonophores dissipate the proton motive force (PMF), a fundamental force that drives physiological functions. We found that E. coli cells responded to protonophores heterogeneously, resulting in bimodal distributions of cell growth, substrate transport, and motility. Furthermore, we showed that this heterogeneous response required active efflux systems. The analysis of underlying interactions indicated the heterogeneous response results from efflux-mediated positive feedback between PMF and protonophores’ action. Our studies have broad implications for bacterial adaptation to stress, including antibiotics.

## INTRODUCTION

An electrochemical proton gradient across the cytoplasmic membrane, alternatively known as proton motive force (PMF), drives vital processes in cells. For example, PMF powers ATP synthesis (1, 2), transport of a wide range of substrates, including essential ions and metabolites (3–6), and motility (7–9). Furthermore, PMF plays an important role in cell division (10) and cell-to-cell signaling (11, 12). Due to its importance, PMF is a key target for chemical warfare between living organisms. For example, bacteria dissipate PMF of other species to increase their colonization (13–18). A host dissipates PMF of pathogens to slow or prevent their invasion (19, 20).

One common way to dissipate PMF is via protonophores. They are a class of ionophores that collapse the proton gradient across the cell membrane by shuffling protons (21–23). Protonophores have been extensively used in various research fields to perturb a wide range of cellular processes, particularly in the antibiotic research field, due to the critical role of PMF in antibiotic influx, efflux, and mechanism of action. For example, aminoglycoside influx is PMF driven (24), and the PMF dissipation by protonophores turns aminoglycoside from bactericidal to bacteriostatic (25) or generates antibiotic-tolerant persisters (26). Furthermore, efflux pumps, a main culprit for multidrug resistance, require PMF to pump antibiotic molecules out of cells (27). Because PMF dissipation has detrimental effects on cells, protonophores themselves work as antibiotic agents (17, 18, 28–33). Perhaps unsurprisingly, protonophores naturally produced by bacteria or hosts alter antibiotic efficacy (20, 34, 35).

The detailed action of protonophores has been extensively studied *in vitro* using reconstituted lipid-bilayer systems (for example, see references 36 and 37). However, *in vivo* effects of protonophores are less well understood, despite the fact that they are critical to bacterial physiology, microbial competition, host-pathogen interaction, and antibiotic action and resistance. In this study, we characterized the cellular response to protonophores.

## RESULTS

### Heterogeneous responses of bacteria to protonophores.

**FIG 1:**
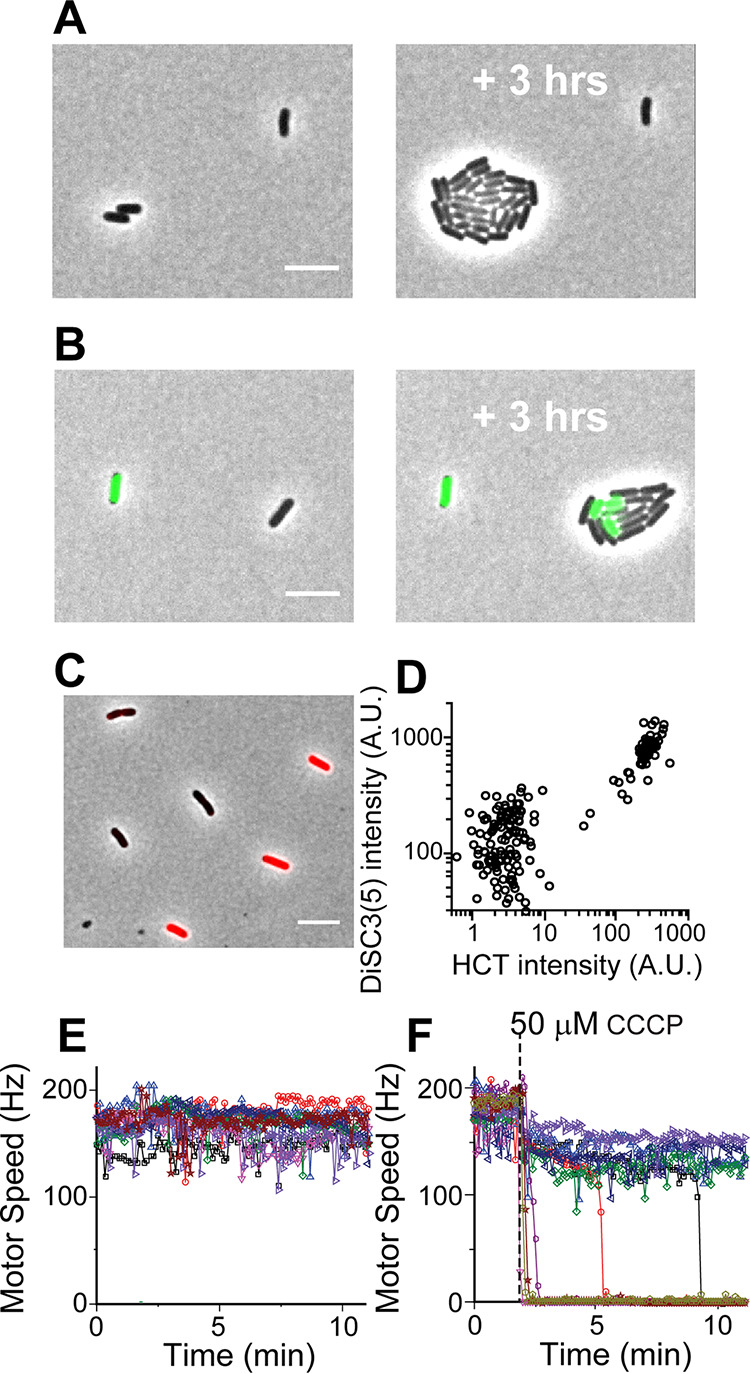
Heterogeneous responses of E. coli to 50 μM CCCP. (A) Some cells grew normally (growth rate of 0.87 ± 0.07/h, which is comparable to 0.83 ± 0.09/h for untreated cells). However, growth was completely inhibited in other cells. (B) Cells exhibited two distinct HCT levels, and HCT-bright cells did not grow. Out of 297 cells, 74 cells were HCT-bright and nongrowing. Cells transitioned from HCT-dim to HCT-bright during the experiment, as indicated by HCT-bright cells in the growing microcolony on the right. (C and D) Cells exhibited two distinct intracellular DiSC_3_(5) intensities, which are correlated with HCT intensities. The scale bar represents 5 μm. Note that HCT intensities are quantified in Fig. 2 and show the HCT intensity distribution differs between the Δ*tolC* and WT strains. Thus, we compared the DiSC_3_(5) intensity in the Δ*tolC* and WT strains. We found that DiSC_3_(5) intensity in the Δ*tolC* strain was moderately higher (∼50%). While this finding agrees with the previous finding that DiSC_3_(5) is a substrate of the efflux pumps (80), the efflux activity is only moderate and cannot explain the 10-fold difference in DiSC_3_(5) intensity between DiSC_3_(5)-bright and DiSC_3_(5)-weak cells in panel D. (E) In the absence of CCCP, the motor speeds were uniform across a population. (F) When exposed to CCCP, cells exhibited two distinct motor speeds.

We measured the growth of E. coli treated with a common protonophore, carbonyl cyanide m-chlorophenyl hydrazine (CCCP). At increasing CCCP concentrations, the rate of population growth decreased gradually (see Fig. S1A in the supplemental material). We then monitored the growth of individual cells at 50 μM CCCP, an intermediate concentration at which a population exhibits a moderate growth reduction (Fig. S1A). We found an all-or-none effect of CCCP at the single-cell level (Fig. 1A); some cells did not grow, whereas other cells continued to grow at the same rate as untreated cells.

10.1128/mBio.00676-21.1 FIG S1 Growth of E. coli under various conditions. (A) Growth curve of WT E. coli treated with various concentrations of CCCP. At 50 μM, the rate of population growth decreased moderately. (B) Growth curve of WT E. coli treated with various concentrations of HCT. At 0.1 μM (the concentration used in the present study), HCT has little effect on cell growth. (C) Growth of WT E. coli treated with 0.1 nM DiSC_3_(5). At this concentration, DiSC_3_(5) has little effect on cell growth. Download FIG S1, TIF file, 0.1 MB. Copyright © 2021 Le et al. 2021 Le et al. https://creativecommons.org/licenses/by/4.0/ This content is distributed under the terms of the Creative Commons Attribution 4.0 International license.

We then examined how CCCP affects substrate transport by using a fluorescent dye, Hoechst 33342 (HCT) (38). Intracellular HCT intensity was uniformly low in the absence of CCCP. At an intermediate CCCP concentration (50 μM), we observed coexistence of cells exhibiting two distinct HCT intensities (Fig. 1B). Importantly, HCT intensity was correlated with cell growth; cells with low intensity (HCT-dim) grew unperturbed, while those with high intensity (HCT-bright) exhibited no growth (Fig. 1B). Some cells transitioned from HCT-dim to HCT-bright during the experiment. After the transition, HCT-bright cells ceased to grow.

We then examined the mechanism for the coexistence of cells with the distinct cell growth and substrate transport states. Given that CCCP disrupts PMF (22), we hypothesized that the effect of CCCP on PMF is heterogeneous, i.e., it disrupts PMF severely in some cells but not in others. To examine this hypothesis, we evaluated PMF using two different approaches. First, we used a dye sensitive to membrane potential, DiSC_3_(5). When extracellular pH is comparable to that of intracellular pH (as is the case for our growth medium), membrane potential is primarily determined by PMF. DiSC_3_(5) accumulates in cells with strong PMF and self-quenches, resulting in low fluorescence (39, 40). We first confirmed that our working DiSC_3_(5) concentration (nanomolars) did not affect cell growth (Fig. S1C). We then exposed cells to 50 μM CCCP and found they exhibited two distinct DiSC_3_(5) intensities (Fig. 1C). Because DiSC_3_(5) and HCT fluorescence emission spectra are well separated, they can be used simultaneously. We found that DiSC_3_(5)-bright cells were also HCT-bright and did not grow (upper right group in Fig. 1D), whereas DiSC_3_(5)-dim cells were HCT-dim and grew (lower left group in Fig. 1D). This observation suggests that the coexistence of cells with two distinct HCT intensities and cell growth states is caused by the heterogeneous effect of CCCP on PMF.

To further test our hypothesis, we next measured the bacterial flagellar motor speed. Flagellar motor is powered by, and its speed is proportional to, PMF (7, 9). By measuring the motor rotation speed, we previously determined a relative change in PMF (41, 42). In the absence of CCCP, the motor speed remained high and constant (Fig. 1E). Treatment with 50 μM CCCP resulted in two subpopulations, one with slightly reduced rotation (high PMF) and the other with no rotation (zero PMF) (Fig. 1F). The time point at which each cell lost PMF varied, agreeing with our observation that cell growth stopped at various times during CCCP treatment (Fig. 1B). Our observation of two distinct motor speeds and DiSC_3_(5) intensities in a population supports our hypothesis that CCCP disrupts the PMF of cells heterogeneously.

**FIG 2:**
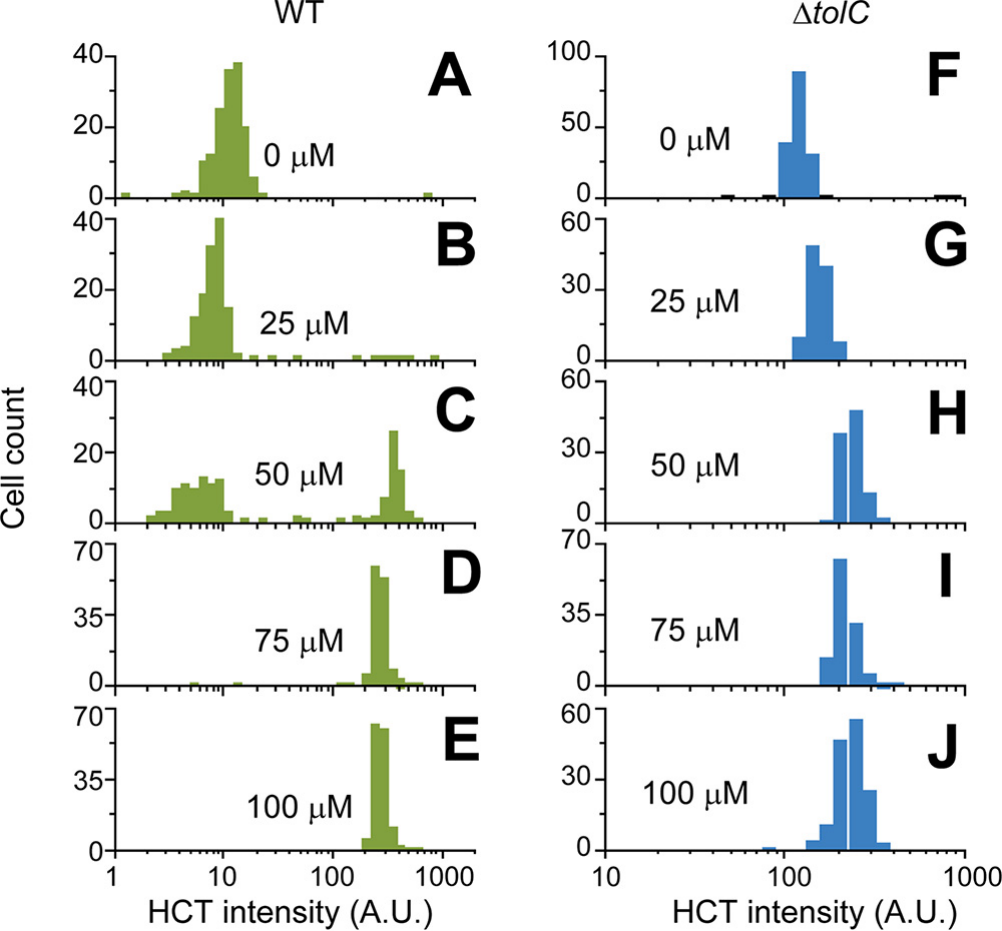
HCT fluorescence intensity distribution in cells treated with various CCCP concentrations. (A and B) At low CCCP concentrations (≤25 μM), HCT intensity was low across a population (HCT-dim), resulting in a unimodal distribution with the peak center near ∼10 AU. (C) Increasing the CCCP concentration to 50 μM did not shift the peak center but led to the appearance of another peak on the right (>100 AU), showing a bimodal distribution. At higher (≥75 μM) CCCP concentrations, the left low-intensity peak disappeared, showing the enrichment of HCT-bright cells. (F to J) The Δ*tolC* strain lacks a peak on the left, exhibiting a unimodal distribution. More than 200 cells were analyzed for each condition. We made a similar observation for two other protonophores, TCS and indole (Fig. S3 and S4).

**FIG 3:**
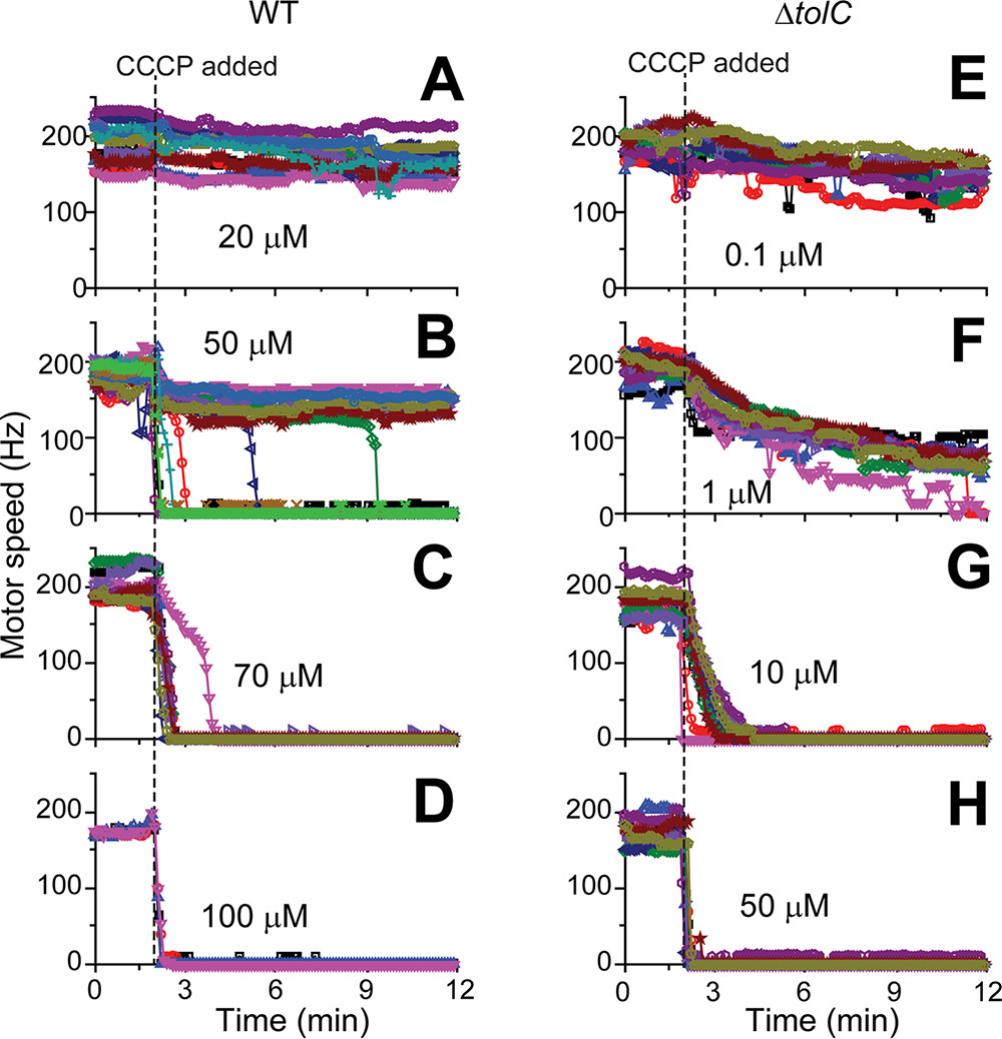
Flagellar motor speeds of cells treated with various CCCP concentrations. (A) At low CCCP concentrations, the motor speeds of WT cells were barely affected. (B) Cells exhibited two distinct motor speeds at 50 μM CCCP, one with a slightly reduced rotation (high PMF) and the other with no rotation (zero PMF). (C and D) At higher CCCP concentrations (≥70 μM), the motor speeds were zero in all cells. (E to H) The Δ*tolC* strain exhibited a uniform and gradual reduction in the motor speed at increasing CCCP concentrations. Note that, at very low PMF values where the bacterial flagellar motor operates with one stator unit, the motor can transiently stop in a step-like manner (81, 82), which can explain the purple trajectory in panel F. Motor speed measurements are laborious and time-consuming. The motor speeds of 10 to 15 cells were analyzed for each condition except for the experiment with the WT strain exposed to 100 μM CCCP (where 4 cells were analyzed).

We then quantified the degree of heterogeneity by characterizing HCT fluorescence intensity and motor speeds over a wide range of CCCP concentrations. At low CCCP concentrations (≤25 μM), HCT intensity in a population was low (HCT-dim) and its distribution was unimodal (Fig. 2A and B). The motor speed was barely affected (Fig. 3A). An increase in the CCCP concentration to 50 μM did not shift the center of the original peak in the HCT intensity distribution but led to the appearance of another peak on the right (HCT-bright cells), showing a bimodal distribution (Fig. 2C). This agrees with the motor speed data, which showed two distinct motor speeds at this concentration (Fig. 3B). At higher CCCP concentrations (≥70 μM), the original peak on the left in the HCT intensity distribution disappeared, indicating the enrichment of HCT-bright cells (Fig. 2D to E). This enrichment is accompanied by the complete collapse of PMFs, as indicated by motor speeds of zero in all cells (Fig. 3C to D).

10.1128/mBio.00676-21.3 FIG S3 HCT intensity distribution in WT (left) and Δ*tolC* (right) cells treated with TCS. (Left) WT strain. At low TCS concentrations (≤5 μM), the HCT intensity was uniformly low across a population, leading to a unimodal distribution (with the peak center around ∼10 AU). Increasing TCS concentrations led to the emergence of the second peak on the right (>100 AU, HCT-bright cells), showing a bimodal distribution. At higher concentrations (≥30 μM), the left low-intensity peak disappeared, showing the enrichment of HCT-bright cells. (Right) Δ*tolC* strain. For all TCS concentrations tested, the left low-intensity peak is absent in the Δ*tolC* strain, which resulted in a unimodal distribution. The center of the single peak shifted moderately at increasing TCS concentrations. This observation is consistent with that made for CCCP (Fig. 2). Download FIG S3, TIF file, 0.2 MB. Copyright © 2021 Le et al. 2021 Le et al. https://creativecommons.org/licenses/by/4.0/ This content is distributed under the terms of the Creative Commons Attribution 4.0 International license.

10.1128/mBio.00676-21.4 FIG S4 HCT intensity distribution in WT (left) and Δ*tolC* (right) cells treated with indole. The result is similar to that from the TCS experiment in Fig. S3. (Left) WT strain. Increasing indole concentrations led to the emergence of the second peak on the right (>100 AU, HCT-bright cells) and a mild shift in the center of the left low-intensity peak. The two peaks merged at 7.5 mM indole concentration. (Right) Δ*tolC* strain. For all indole concentrations tested, a left low-intensity peak is absent in the Δ*tolC* strain. The center of the single peak shifted moderately at increasing indole concentrations. Of note, we previously measured the motor speeds of WT cells at low indole concentrations (≤2 mM), which showed a uniform reduction in the motor speeds (41). Here, we observed a bimodal distribution of the HCT intensity at higher concentrations (≥2.5 mM). At 2.5 mM, the fraction of cells exhibiting high HCT intensities was less than 5%, which is difficult to detect with motor speed measurements because motor speed measurements are laborious, and it is challenging to track more than 20 cells. Download FIG S4, TIF file, 0.2 MB. Copyright © 2021 Le et al. 2021 Le et al. https://creativecommons.org/licenses/by/4.0/ This content is distributed under the terms of the Creative Commons Attribution 4.0 International license.

### The heterogeneous effect of a protonophore is mediated by the efflux pumps.

**FIG 4:**
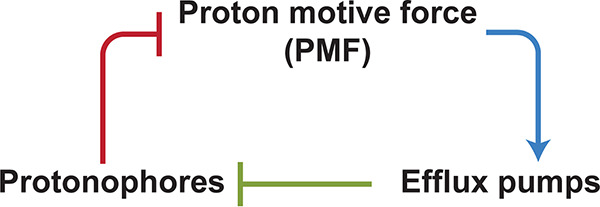
Model of efflux-mediated positive feedback. Efflux pumps transport protonophores out of cells (44, 45) (green arm). The pump is powered by PMF (27) (blue arm) and, thus, is subject to disruption by protonophores (red arm).

Our observations described above confirm that cells exposed to CCCP exhibit distinct PMF levels. In bacteria, positive feedback is required to stabilize distinct phenotypic states (43). Here, we investigated a feedback mechanism that stabilizes two distinct PMF levels in a CCCP-exposed population. Bacteria can mitigate harmful effects of protonophores and other toxic compounds by extruding them with efflux pumps (44–46) (green arm in Fig. 4). However, these pumps are powered by PMF (27) (blue arm in Fig. 4) and, thus, are subject to disruption by protonophores (red arm in Fig. 4), suggesting an efflux-mediated positive feedback between protonophores and PMF (Fig. 4).

We experimentally tested this potential role of efflux activity by repeating our measurements using the Δ*tolC* strain. In many bacterial species, including E. coli, TolC is a major component of efflux pumps (47), and these pumps can be inactivated by the *tolC* knockout. We first confirmed that the *tolC* knockout itself had little effect on the PMF level in the absence of CCCP (Fig. S2). Our HCT measurements showed that the HCT intensity was uniform across a Δ*tolC* population, and the analysis showed the absence of a left, low-intensity peak, which results in a narrow unimodal distribution (Fig. 2F). Increasing CCCP concentrations moderately shifted the peak center, but the distribution remained unimodal (Fig. 2F to J), which is in contrast to a bimodal distribution in the wild-type (WT) strain (Fig. 2A to E). This observation with the Δ*tolC* strain is consistent with motor speed measurements, which showed that Δ*tolC* cells exhibited a uniform and gradual reduction in the motor speed at increasing CCCP concentrations (Fig. 3E to H). These data indicate that the efflux pumps indeed play a critical role in the heterogeneous effect of a protonophore.

10.1128/mBio.00676-21.2 FIG S2 Motor speeds of WT (green) and Δ*tolC* (blue) cells in the absence of protonophores. Here, we compared the motor speeds of WT and Δ*tolC* strains. Their motor speeds were comparable, indicating that Δ*tolC* has no noticeable effect on the PMF. Download FIG S2, TIF file, 0.1 MB. Copyright © 2021 Le et al. 2021 Le et al. https://creativecommons.org/licenses/by/4.0/ This content is distributed under the terms of the Creative Commons Attribution 4.0 International license.

We next examined the HCT intensity distribution in cells treated with other common protonophores, 3,3′,4′,5-tetrachlorosalicylanilide (TCS) (48, 49) and indole (37, 41). Similar to CCCP, the WT cells exhibited two distinct HCT intensities in intermediate concentrations of these protonophores, and the analysis confirmed a bimodal distribution (Fig. S3 and S4, left). The centers of the two peaks were comparable to those observed in a CCCP-treated population, the left peak at ∼10 arbitrary units (AU) and the right peak at >100 AU. In a Δ*tolC* population, however, the HCT intensity was uniform, and the analysis showed the absence of a left, low-intensity peak (Fig. S3 and S4, right), which is consistent with our finding from the experiment with CCCP.

## DISCUSSION

PMF is at the basis of vital physiological functions in cells (1–11). Protonophores are synthesized for a research purpose or produced naturally by living organisms (13–20). For example, indole (a protonophore [37, 41] tested in the present study) is one of the most abundant compounds in a dense bacterial culture and present in high concentrations in gut microbiome (12, 50). Protonophores collapse the total PMF that acts on protons by allowing them to equilibrate (22, 37, 51). Our additional analysis of the functional dependence of the motor speed on the CCCP concentration (see Fig. S5 in the supplemental material) is consistent with this known mechanism of action.

10.1128/mBio.00676-21.5 FIG S5 Our results agree with the known CCCP action as a protonophore. Previous *in vitro* measurements have showed that CCCP collapses the total PMF that acts on protons by allowing them to equilibrate (22, 37). To confirm this mechanism of CCCP action, we analyzed a relative change in the motor speed as a function of a CCCP concentration, [CCCP], in the Δ*tolC* strain: *ω/ω_0_ =*PMF/PMF_0_ *= f* ([CCCP],*t*), as we have shown before (41). The mean speeds during the last 2 min of CCCP exposure (Fig. 3E to H) were obtained from Gaussian fits to probability densities of speeds left) and normalized to the pre-CCCP treatment speed. We then fit the normalized speeds with a hyperbolic function and obtained the following relationship, PMF/PMF_0_ = 1/(2.64 × [CCCP]*+*1) (right). This agrees with an inverse relationship between PMF and [CCCP] from *in vitro* studies (22, 37), supporting that CCCP acts as a protonophore. We note that, unlike some other ionophores, such as butanol and indole (41), the PMF reduction at a given CCCP concentration takes several minutes (Fig. 3E to H). Download FIG S5, TIF file, 0.4 MB. Copyright © 2021 Le et al. 2021 Le et al. https://creativecommons.org/licenses/by/4.0/ This content is distributed under the terms of the Creative Commons Attribution 4.0 International license.

Efflux pumps transport protonophores out of cells, protecting them from protonophores’ harmful effects (44, 45). The present study demonstrates that this protection is heterogeneous, protecting some cells but not all. Our findings indicate that this heterogeneity emerges because protonophores affect their own efflux transport. For example, if cells initially have a strong efflux activity, upon the exposure to protonophores, they extrude protonophores better and maintain their PMF, thereby continuing to support the strong efflux activity (opposite for cells with weak efflux activity).

PMF has important roles in antibiotic influx, efflux, and mechanism of action. As such, protonophores were extensively used in antibiotic research. Interestingly, heterogeneous responses were observed in these studies. For example, a subpopulation of cells can tolerate antibiotics by not growing: bacterial persisters (52, 53). The postantibiotic effect, continued growth suppression after antibiotic withdrawal, is strongly skewed by a small subpopulation that resumes growth earlier than others (54). Protonophores have strong effects on the emergence of a persister subpopulation (26, 55) and an early-grower subpopulation (56). Importantly, these heterogeneous effects of protonophores involve efflux pumps (26, 55–57). This is consistent with our observation of heterogeneous growth phenotypes (i.e., the coexistence of nongrowing and growing subpopulations), which is mediated by the feedback among protonophores, PMF, and efflux pumps. Our additional motor-speed data indicate that, upon protonophore washout, cells in the nongrowing subpopulation recover their PMFs heterogeneously (Fig. S6). We believe that our findings are useful for antibiotic research, given the important role of PMF and efflux pumps to antibiotic action and widespread use of protonophores in the field. Furthermore, some protonophores are used as antibiotic agents (17, 18, 28–33), for which our findings can be directly applicable.

10.1128/mBio.00676-21.6 FIG S6 Recovery of PMF after CCCP washout. We have discussed in the main text that the treatment with 50 μM CCCP resulted in two subpopulations, one with a moderate reduction in the motor speed and the other with a motor speed of zero. When CCCP was removed, the former recovered the motor speed immediately. Partial recovery was observed in some cells in the latter subpopulation (red arrow). Download FIG S6, TIF file, 0.1 MB. Copyright © 2021 Le et al. 2021 Le et al. https://creativecommons.org/licenses/by/4.0/ This content is distributed under the terms of the Creative Commons Attribution 4.0 International license.

Our results have broad implications for bacterial adaptation to stress. In natural environments, various toxic compounds negatively affect bacterial physiology. While bacteria harbor defense mechanisms to mitigate the toxicity, these mechanisms are often coupled to the physiological state of the cells and become ineffective when the physiology is severely disturbed. In our studies, this coupling is manifested as the feedback between protonophores and efflux pumps. Similar coupling could be realized through other mechanisms. For example, efflux pumps extrude biocides or other plant-derived disinfectants, but their expression is altered by these compounds, thereby forming feedback (58, 59). Cytoplasmic pH is an important determinant for antibiotic efflux, as it contributes to PMF and can affect the expression of some efflux systems (60, 61). However, pH can be altered by antibiotics (51, 62) (e.g., nigericin specifically targets the cytoplasmic pH), suggesting an additional feedback mediated by pH. Our studies provide insight into how such coupling could affect bacterial adaptation to these toxic compounds.

Lastly, protonophores have been extensively utilized as a powerful tool to perturb various physiological processes in cells, including cell division, motility, and antibiotic transport. It was commonly assumed that increasing protonophore concentrations lead to gradual disruption of these processes. However, our studies confirm that the disruption is heterogeneous at the single-cell level. Our data from the experiment with the Δ*tolC* strain show that gradual disruption can be achieved but requires the inactivation of efflux activities. Additionally, we provide a functional dependency of the PMF loss on a CCCP concentration (Fig. S5), which can facilitate the use of CCCP to perturb PMF in a quantitative manner. These results should be useful for experimental designs and data interpretation in future studies.

## MATERIALS AND METHODS

### Bacterial strains and growth conditions.

E. coli K-12 NCM3722 (63–65) and Neidhart’s morpholine propanesulfonic acid (MOPS) minimal medium (66) with glucose and ammonium as the carbon and nitrogen sources were used, except for the motor speed measurement (see below). See Table S1 in the supplemental material for all the ingredients and their concentrations used in the media. The medium pH is 7.0, which allowed E. coli to keep neutral cytoplasmic pH (67–69) and ensured HCT fluorescence does not incur any pH-related intensity changes (70). To make the Δ*tolC* strain (NMK320), the *tolC* gene deletion allele from the Keio deletion collection (71, 72) was transferred to the NCM3722 strain using P1 transduction (73). The Km^r^ gene was flipped out as previously described (71, 72).

10.1128/mBio.00676-21.7 TABLE S1 Chemicals used in our experiments. Download Table S1, DOCX file, 0.02 MB. Copyright © 2021 Le et al. 2021 Le et al. https://creativecommons.org/licenses/by/4.0/ This content is distributed under the terms of the Creative Commons Attribution 4.0 International license.

Cells were cultured at 37°C with constant agitation at 250 rpm in a water bath (New Brunswick Scientific). To monitor their growth, the optical density at 600 nm (OD_600_) of the culture was measured using a Genesys20 spectrophotometer (Thermo-Fisher) with a standard cuvette (16.100-Q-10/Z8.5; Starna Cells Inc.). To prepare experimental cultures, cells were taken from −80°C stocks and first grown in 5 ml LB medium at an OD_600_ of ∼0.001 (seed culture). At an OD_600_ of ∼0.5 (before cells entered stationary phase), we resuspended cells in 5 ml minimal growth medium at very low densities (typically lower than an OD_600_ of ∼0.0001) and cultured them overnight (preculture). The next morning, the preculture was diluted in prewarmed, 5 ml minimal growth medium (experimental culture) to an OD_600_ of ∼0.01 (20 to 50 times dilution) and allowed to grow exponentially to an OD_600_ of ∼0.1, at which measurements were performed.

### Fluorescence microscopy.

To make fluorescence measurements, cells were treated with protonophores at an OD_600_ of 0.1 for 10 min and then with HCT and/or DiSC_3_(5) for 60 min at 37ºC in constant agitation in the dark; 0.1 μM HCT and 0.1 nM DiSC_3_(5) were sufficient to produce discernible intracellular fluorescence signals while not affecting cell growth (Fig. S1). The cells then were loaded onto no. 1.5 cover glasses; 1-mm-thick 1.5% agarose pads, made with the same MOPS growth medium [containing the same concentrations of HCT, DiSC_3_(5) and/or protonophores], were used to cover the cells.

Cells were imaged with a prewarmed (at 37°C) inverted microscope (Olympus IX83 P2Z) with a Neo 5.5 sCMOS camera (Andor Neo). Intracellular HCT and DiSC_3_(5) were imaged using 4′,6-diamidino-2-phenylindole (DAPI) and Cy5 fluorescence filter sets. Images were acquired with MetaMorph Microscopy Automation and Image Analysis Software and analyzed with the MicrobeJ 5.13m_4_ plug-in in ImageJ (74). MicrobeJ can automatically segment cell boundaries from phase-contrast microscope images and apply the binary masks from the segmentation to measure fluorescence intensities inside and outside the cells. The latter (background) is subtracted from the former to determine intracellular fluorescence signals.

### Motor speed measurement.

E. coli K-12 MG1655 with genetically modified flagellar filaments (EK07 [41]) was used. It was cultured in lysogeny broth (LB) (10 g tryptone, 5 g yeast extract, 10 g NaCl per 1 liter) to an OD_600_ of ∼2. Cells were sheared to truncate flagellar filaments, washed from LB to modified minimal medium (MM9; 50 mM Na_2_HPO_4_, 25 mM NaH_2_PO_4_, 8.5 mM NaCl, 18.7 mM NH_4_Cl, 0.1 mM CaCl_2_, 1 mM KCl, 2 mM MgSO_4_, pH 7.5) supplemented with 0.3% d-glucose and attached to the cover glass surface of a tunnel slide via poly-l-lysine (41, 75, 76). We have shown that the cytoplasmic pH of the cells in this medium is ∼7.8 (41). Based on these values (medium pH 7.5 and cytoplasmic pH 7.8), we estimate that the maximum contribution of ΔpH to the PMF under our condition is <30mV (41). Polystyrene beads (0.5 μm) were attached to truncated flagellar filaments and placed into the focus of a heavily attenuated optical trap (855-nm laser) to detect the motor rotation (41). Time course of the bead rotation was recorded with the position-sensitive detector (model 2931; New Focus, Irvine, CA) at 10 kHz, and a 2.5-kHz cutoff antialiasing filter was applied before processing the signal. Next, a flat-top window discrete Fourier transform (window size, 16,384 data points with a step dt of 0.01 s) was applied to the acquired *x* and *y* coordinates of a bead position to obtain a time series motor speed recording. The speed traces were then median filtered with a 401-point moving window after manual removal of spurious zeroes caused by flowing CCCP/medium into the slide. The filtered speed trace was then resampled to 10 samples/min for plotting. Measurements were made with a microscope equipped with back focal plane interferometry capability (77, 78), as described previously (77–79).
