# Supplementary figures and images for: Active Efflux Leads to Heterogeneous Dissipation of Proton Motive Force by Protonophores in Bacteria
Source: mBio. 2021 Jul 13;12(4):e00676-21. doi: 10.1128/mBio.00676-21 (PMC8406135; doi:10.1128/mBio.00676-21)

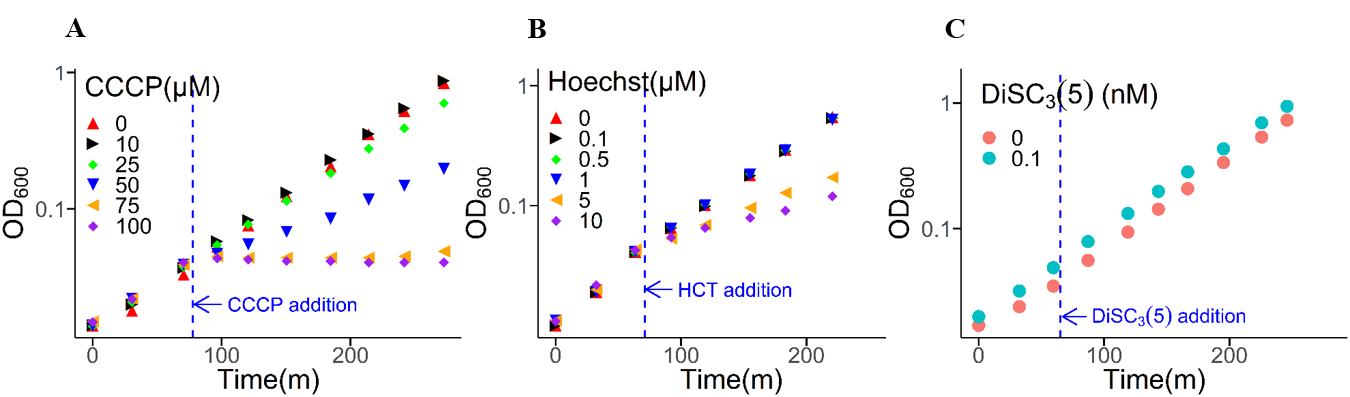

Supplement: FIG S1 [file mbio.00676-21-sf001.tif]

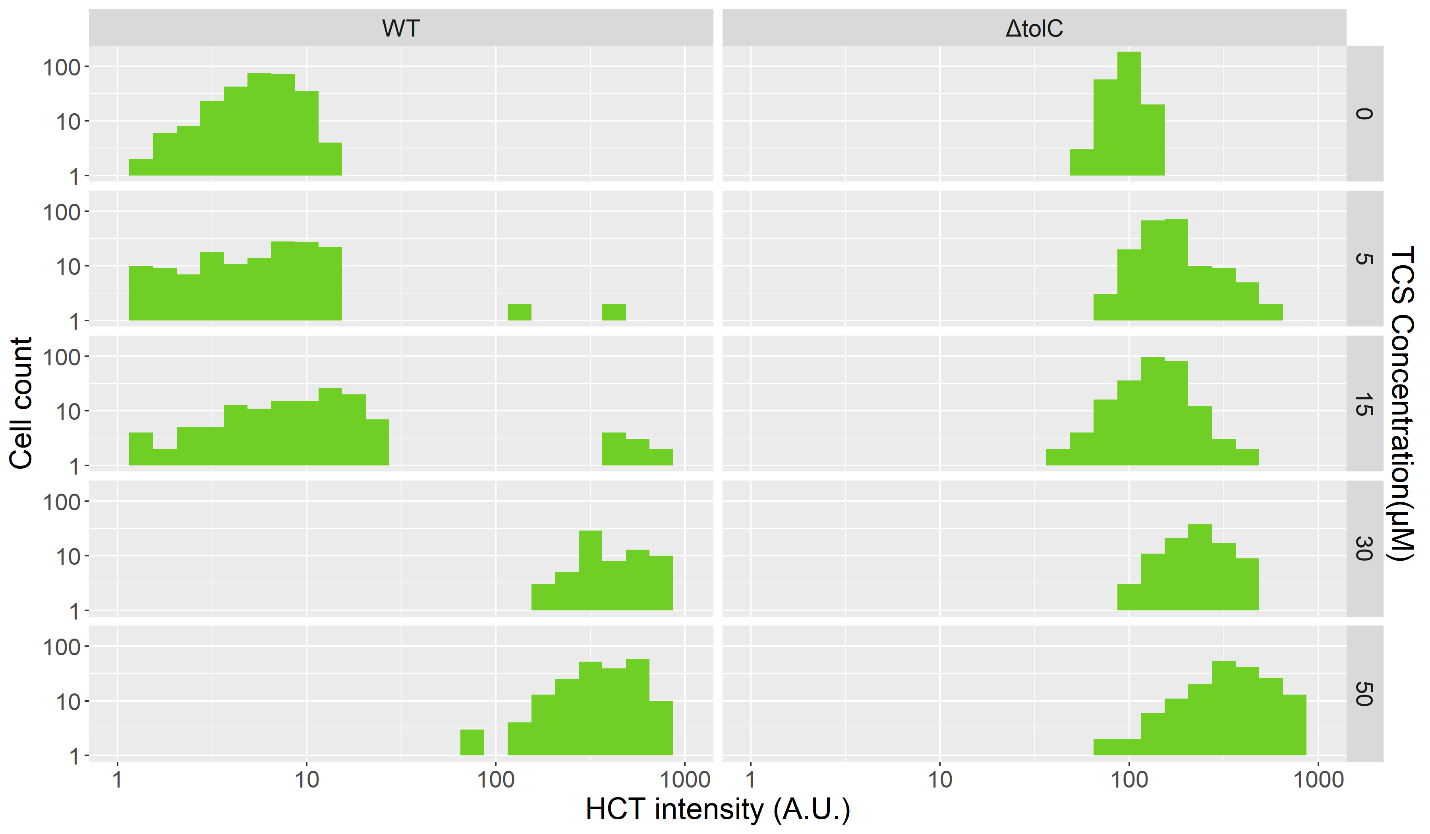

Supplement: FIG S3 [file mbio.00676-21-sf003.tif]

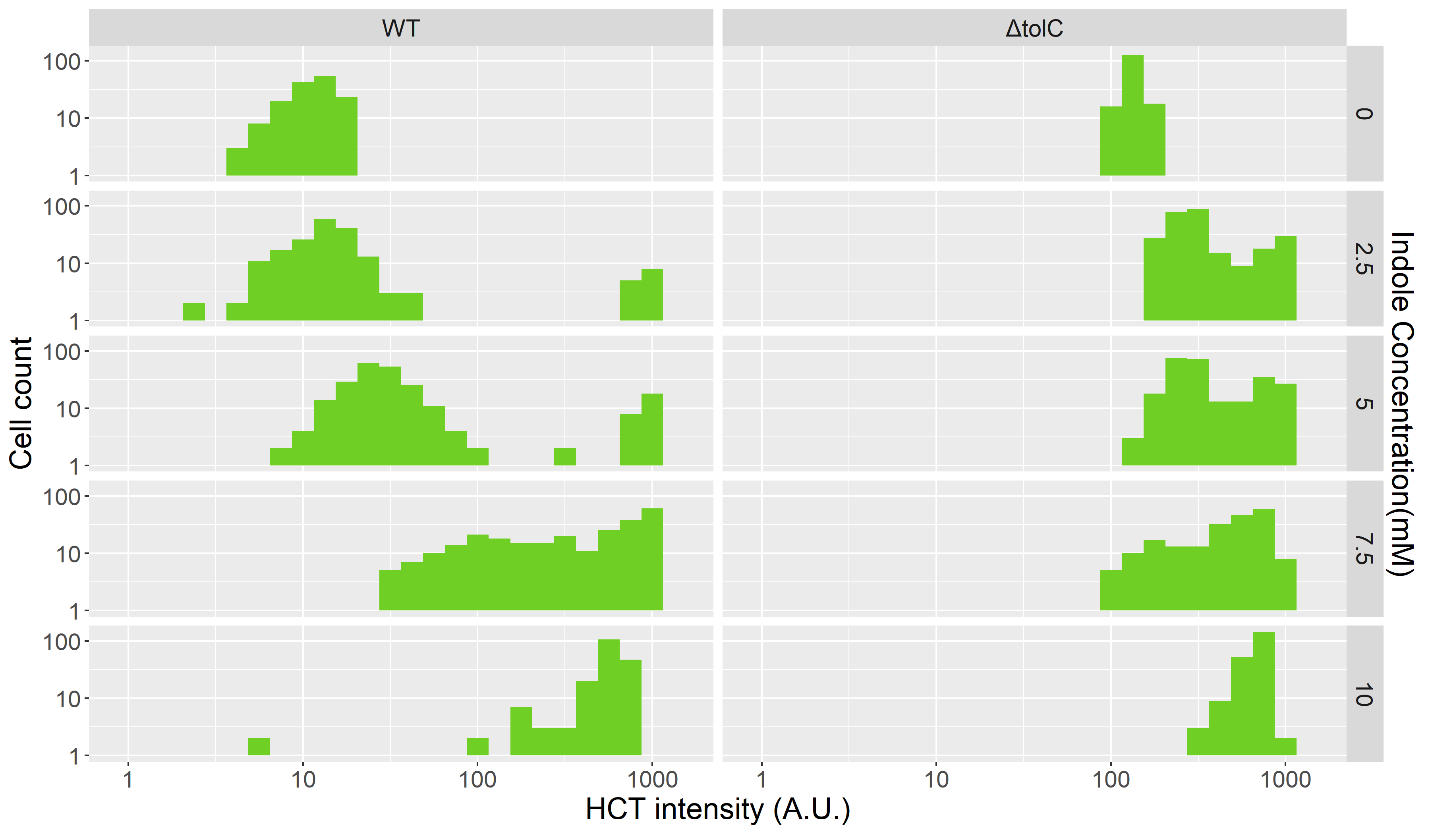

Supplement: FIG S4 [file mbio.00676-21-sf004.tif]

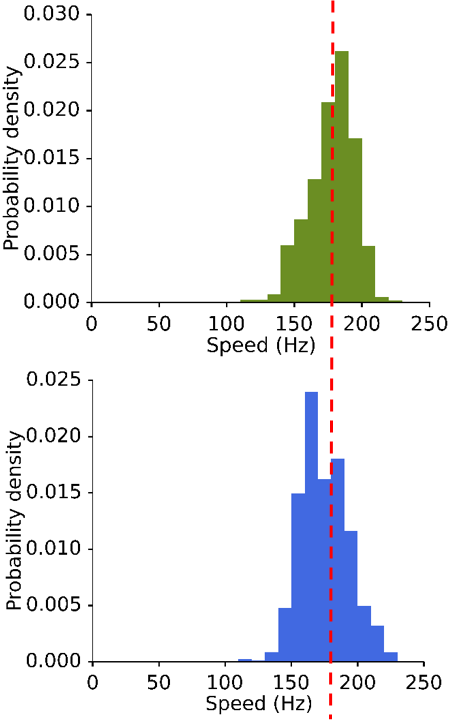

Supplement: FIG S2 [file mbio.00676-21-sf002.tif]

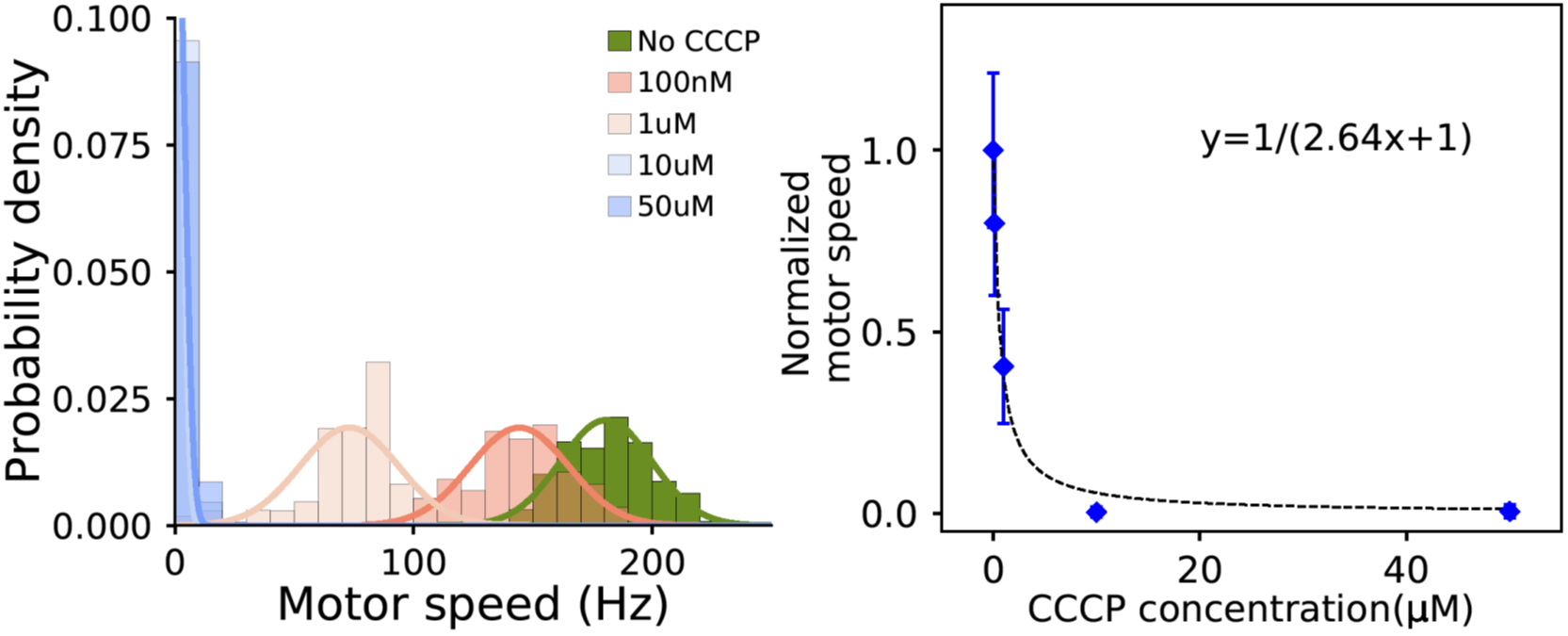

Supplement: FIG S5 [file mbio.00676-21-sf005.tif]

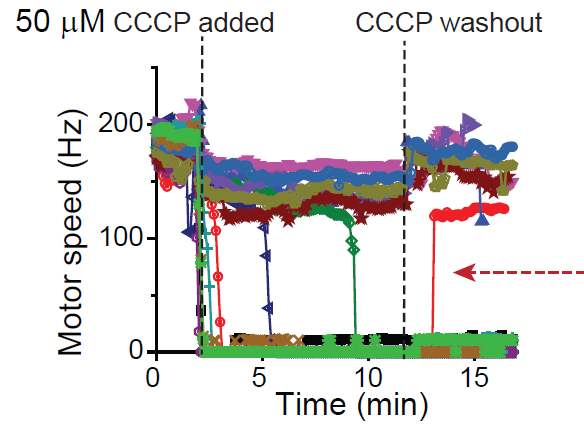

Supplement: FIG S6 [file mbio.00676-21-sf006.tif]
