# Supplementary material for: Active Efflux Leads to Heterogeneous Dissipation of Proton Motive Force by Protonophores in Bacteria
Source: mBio. 2021 Jul 13;12(4):e00676-21. doi: 10.1128/mBio.00676-21 (PMC8406135; doi:10.1128/mBio.00676-21)
Supplement: TABLE S1 [file mbio.00676-21-st001.docx]

**Supplementary table**

**Materials**

Ingredients used in the MOPS minimal media are provided below.

**Supplementary Table 1A**

| Ingredient | Concentration | Manufacturer | Catalog |
| --- | --- | --- | --- |
| **MOPS** | 1 mM | Sigma Aldrich | 1132-61-2 |
| **Tricine** | 1 mM | Sigma Aldrich | 5704-04-1 |
| **FeSO_4_** | 0.01 mM | Acros Organics | 7782-63-0 |
| **K_2_SO_4_** | 0.276 mM | Sigma Aldrich | 7778-80-5 |
| **CaCl_2_** | 0.05 mM | Sigma Aldrich | 10043-52-4 |
| **MgCl_2_.6H_2_O** | 0.523 mM | Sigma Aldrich | 7791-18-6 |
| **K_2_HPO_4_** | 0.132mM | Sigma Aldrich | 7758-11-4 |
| **NaCl** | 50 mM | Sigma Aldrich | 7647-14-5 |
| **NH_4_Cl** | 10mM | Sigma Aldrich | 12125-02-9 |
| **Glucose** | 10mM | Sigma Aldrich | 50-99-7 |
| **Micronutrients** | | | |
| (NH_4_)_6_Mo_7_O_24_*4H_2_O | 3 nM | Acros Organics | 12054-85-2 |
| H_3_BO_3_ | 400 nM | Milipore | 10043-35-3 |
| CoCl_2._6H_2_O | 30 nM | Acros Organics | 7791-13-1 |
| CuSO_4_ | 10 nM | Acros Organics | 7758-99-8 |
| MnCl_2_ | 80 nM | Acros Organics | 13446-34-9 |
| ZnSO_4._7H_2_O | 10 nM | Acros Organics | 7446-20-0 |

Other chemicals used in the experiments

**Supplementary Table 1B**

| Chemical | Manufacturer | Catalog |
| --- | --- | --- |
| LB broth (Miller) | Fisher Scientific | 12795027 |
| Carbonyl cyanide 3-chlorophenylhydrazone (CCCP) | Sigma Aldrich | 555-60-2 |
| 3,3',4',5-Tetrachlorosalicylanilide (TCS) | Acros Organics | 1154-59-2 |
| Indole | TMO | A14427-18 |
| bisBenzimide Hoechst 33342 trihydrochloride | Sigma Aldrich | 875756-97-1 |
| DiSC_3_(5) (3,3'-Dipropylthiadicarbocyanine Iodide) | Milipore Sigma | 53213-94-8 |
